# High-Entropy Alloy Activating Laves-Phase Network for Multi-Component Metallic Coatings with High Hardness

**DOI:** 10.3390/nano14121016

**Published:** 2024-06-12

**Authors:** Ao Yan, Guoxing Chen, Huiqiang Ying, Xiao Yang, Zongde Kou, Song Tang, Longlong Fan, Xiang Chen, He Zhu, Zhiguang Zhu, Yang Ren, Si Lan

**Affiliations:** 1 Herbert Gleiter Institute of Nanoscience, School of Materials Science and Engineering, Nanjing University of Science and Technology, Nanjing 210094, China; 2 Institute of High Energy Physics, Chinese Academy of Sciences, Beijing 100049, China; 3 Nano and Heterogeneous Materials Center, School of Materials Science and Engineering, Nanjing University of Science and Technology, Nanjing 210094, China; 4 School of Mechanical Engineering, Nanjing University of Science and Technology, Nanjing 210094, China; 5 Department of Physics, City University of Hong Kong, Hong Kong 999077, China

**Keywords:** high-entropy alloy, Laves phase strengthening, additive manufacturing

## Abstract

The low hardness and poor wear resistance of laser-cladding 316L stainless steel impose significant constraints on its practical applications. In this study, a strategy for strengthening laser-cladding 316L stainless steel with WMoTaNb refractory high-entropy alloy as a reinforcement material is proposed. The results confirm that the coating primarily comprises a body-centered cubic (BCC) Fe-based solid solution, a network-distributed hexagonal Fe_2_X (X = W, Mo, Ta, and Nb) Laves phase, and a diffusely distributed face-centered cubic (FCC) (Ta, Nb)C phase. The Fe-based solid solution distributes along columnar and fine dendrites, while the Laves phase and (Ta, Nb)C phase are in the inter-dendrites. The presence of a significant number of network Laves phases exhibiting high strength and hardness is the primary factor contributing to the enhancement of coating microhardness. The hardness of the composite coating is increased by nearly twice compared to that of the 316L coating, resulting in an improved wear resistance. The present work can shed light on designing and fabricating 316L stainless steel coating with enhanced hardness and wear resistance.

## 1. Introduction

316L stainless steel is a workhorse material in modern industry due to its good mechanical properties [1,2,3], excellent corrosion resistance [4,5], and low cost. Therefore, it is extensively employed as a corrosion-resistant coating material in various fields such as marine engineering, aerospace engineering, nuclear engineering, and so on [6,7,8]. The current methods for fabricating surface coatings include plasma spraying [9], magnetron sputtering [10], and laser-cladding [11]. Among these methods, laser-cladding technology as a kind of additive manufacturing has the advantages of good flexibility, good metallurgical bonding, and a low dilution rate [12]. In practical applications, it can fabricate uniform and dense high-performance alloy coatings that meet certain thickness requirements [13]. Many researchers are investigating the laser-cladding of 316L stainless steel coatings [14,15]. Zhuang et al. [11] fabricated 316L coatings on 45 steel substrates by coupling the laser-cladding and ultrasound. The application of ultrasonic assistance increased the geometrically necessary dislocation density in the 316L coating, which promoted its average hardness from 199.1 HV to 207.9 HV. Chen et al. [16] investigated the effect of solution treatment on the wear resistance of laser-cladding 316L stainless steel. The carbide particles formed during laser-cladding decompose and the elements subsequently dissolve into the austenite matrix after the solution temperature (1050 °C), resulting in solid solution strengthening. The results indicate that the microhardness and wear resistance of the coating increased after solution treatment. The average microhardness measures 240 HV_0.3_. However, the low hardness and poor wear resistance of 316L stainless steel restrict its broader application [17,18].

To enhance the surface hardness and poor wear resistance, the addition of cemented carbide to fabricate 316L stainless steel matrix composite coatings has proved to be an efficient method [19,20,21]. Ho et al. [22] found that the TiC and NbC ceramic particles were distributed at the grain boundaries of laser-cladding 316L stainless steel, which effectively inhibited grain growth and refined the microstructure, enhancing the microhardness. Wang et al. [23] prepared VC-Cr_7_C_3_ metal matrix composite (MMC) coatings on structural steel substrates by premixing vanadium, carbon, and 316L stainless steel powders using laser-cladding technology. The microhardness of the coatings was significantly increased from 207 HV_1_ to 467.8 HV_1_ when the total content of V and C was increased to 14 wt.%. The carbides, however, exhibit inadequate interfacial compatibility with the stainless steel substrate compared to the metal matrix reinforcement materials, resulting in poor bonding. Thus, it is necessary to explore novel reinforcement materials to enhance the bonding behavior with 316L stainless steel.

In recent years, the emergence of high-entropy alloys (HEAs) has presented novel solutions for the aforementioned issues. HEAs are novel alloys consisting of five or more major elements with excellent properties, such as high hardness, excellent oxidation resistance, and good high-temperature stability [24,25,26]. For example, the face-centered cubic (FCC) structure of Cantor alloys (CoCrFeMnNi) [27], the body-centered cubic (BCC) structure of TiZHfNbTa alloy [28], and other high-entropy alloy systems have been extensively investigated [29,30,31,32,33]. Among the various HEA systems, WMoTaNb and WMoTaNbV refractory HEAs (RHEAs) developed by Senkov et al. [34,35] have attracted significant attention due to their high hardness, high-temperature resistance, and wear resistance. Li et al. [36] fabricated WMoTaNb RHEA coatings on Inconel 718 superalloy substrates using laser-cladding coupled with ultrasound. With the introduction of the ultrasound, the microstructure of the coating became uniformly distributed, and the average grain size decreased, resulting in an increase in hardness from 753 HV_0.5_ to 980 HV_0.5_. Jiang et al. [37] fabricated defect-free WMoTaNb RHEA coatings on 45 steel using a laser-cladding technique. Although the substrate diluted the coating heavily, the surface hardness was 551 HV_0.2_, much higher than the substrate (~320 HV_0.2_). As a result, the significant potential of RHEAs as a novel reinforcement material for enhancing the hardness and wear resistance of austenitic stainless steel cannot be overlooked. It is anticipated that the RHEAs and 316L stainless steel can be utilized to fabricate metal matrix composites featuring excellent metallurgical bonding and superior comprehensive mechanical performance.

This work proposes a strategy for fabricating the WMoTaNb RHEAs/316L metallic composite coatings by laser-cladding. The 316L exhibits excellent metallurgical bonding compatibility with the Fe-based substrate, while simultaneously enhancing the mechanical properties through the incorporation of RHEAs. The WMoTaNb/316L multi-component metallic composite coating was fabricated, and its microstructure evolution was investigated. The effect of WMoTaNb RHEA on the mechanical properties of composite coating is further discussed. The present work is a valuable guide for exploring the feasibility of utilizing RHEAs as reinforcement materials in stainless steel systems.

## 2. Experimental Procedures

### 2.1. Materials

The substrate for laser-cladding was a 45 steel plate with dimensions of 100 mm × 100 mm × 15 mm. Before laser-cladding, the substrate surface was polished using 2000 mesh sandpaper followed by additional polishing and subsequently cleaned with alcohol. The WMoTaNb HEAs and 316L stainless steel powders were supplied by Beijing Yijin New Material Technology Co. (Beijing, China), the composition of which is listed in Table 1. The mass ratio of WMoTaNb refractory high-entropy alloy to 316L stainless steel mixed powders was 3:7, considering the combination of laser energy and the melting point of the raw materials. The experiments were carried out using a coaxial powder-feeding laser system (2000 W Laserline) under argon protection to prepare the laser-cladding samples. The laser-cladding parameters were determined as follows: laser power of 1900 W, scanning speed of 10 mm/s, spot diameter of 2.5 mm, and lap rate of 50%. The wear rate (*W*) was calculated using the following equation:

(1) $$W=\frac{V}{L\times D}$$

where *V* is the negative volume (mm^3^), *D* denotes the normal load (N), and *L* represents the sliding distance (mm).

### 2.2. Microstructure Characterization

The laser-cladding samples were cut down by electrical discharge machining (EDM) with wires perpendicular to the substrate, and the cross-sections of the sample underwent mechanical polishing using sandpaper of various mesh sizes (400, 800, 1200, 1500, and 2000), followed by preliminary polishing with special sandpaper. Subsequently, a sequence of diamond-suspended polishing liquids (3 μm, 1 μm, and 0.5 μm) was used on a polishing cloth attached to an automatic grinding and polishing machine to achieve a mirror finish for scanning electron microscope (SEM) observation. The microstructure characterizations were conducted using a SEM (JSM-7800F PRIME, JEOL Ltd., Tokyo, Japan) at a voltage of 10 kV. The samples were mechanically polished and then thinned by an ion beam (GATAN PIPS II 695, Gatan Inc., Pleasanton, CA, USA) for transmission electron microscopy (TEM) observation. TEM (JEOL JEM-2100F, JEOL Ltd., Tokyo, Japan) equipped with energy dispersive X-ray spectrometry (EDS Oxford Instruments X-Max 80T, Oxford Instruments, Abingdon, UK) was used to characterize the phase structure of coatings further at a voltage of 200 kV. The phase structure of 316L and WMoTaNb powders was measured by high-energy synchrotron X-ray diffraction (S-XRD), which was conducted at the beamline BL03HB of the Shanghai Synchrotron Radiation Facility (SSRF), Shanghai Advanced Research Institute, Chinese Academy of Sciences. The beam size of a high-energy X-ray of wavelength 0.6199 Å was 50 µm × 50 µm, and the energy was 20 keV. The phase structure of the coatings was measured by S-XRD, which was conducted at the beamline 3W1 of the Beijing Synchrotron Radiation Facility (BSRF), Institute of High Energy Physics, Chinese Academy of Sciences. The beam size of high-energy X-ray of wavelength 0.2061 Å was 139 µm × 139 µm, and the energy was 60 keV.

### 2.3. Microhardness and Wear Behavior Tests

The microhardness of the coating from the surface to the substrate was measured by a digital Vickers microhardness tester (HVS-1000A, HST Group, Jinan, China), with a test load of 300 g and an indentation time of 15 s. Each test site was spaced at an interval of 0.2 mm, and each datum was tested five times to obtain the average value and standard error. Nanoindentation tests were performed on an Agilent Nano Indenter G200 (Agilent Technologies, Santa Clara, CA, USA) with a Berkovich diamond tip at room temperature. The nanoindentation hardness of the samples were characterized under a constant holding load F of 10 mN with a constant loading rate of 1 mN/s. The position of the nanoindentation experiment on the coating cross-section was selected and tested based on a 5 × 10 array. Then, the indentation morphology was observed using a SEM and connected with the results of nanoindentation experiments one by one. The wear resistance was evaluated using a linear reciprocating friction and wear tester (Bruker UMT-2, Billerica, MA, USA), with each sample tested twice for accuracy. The test configuration consisted of an Al_2_O_3_ friction pair with a 4 mm diameter, applying a load of 15 N. The length of the wear track was set to 1 mm, and the reciprocating speed was maintained at 4 mm/s throughout the 60 min test duration at room temperature. The surface of the samples was polished to a mirror finish prior to the friction and wear tests, in a procedure consistent with SEM sample preparation.

## 3. Results and Discussions

### 3.1. Phase Analysis

**Figure 1:**
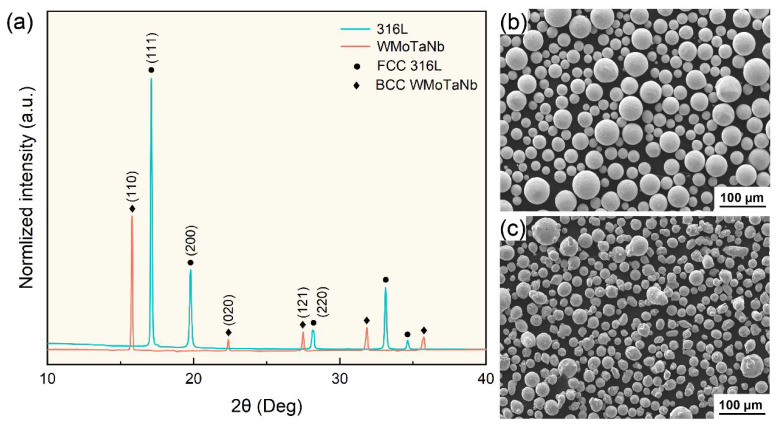
S–XRD patterns and SEM images of 316L stainless steel and WMoTaNb alloy powders. (**a**) S–XRD patterns, (**b**) SEM image for WMoTaNb, (**c**) SEM image 316L.

**Figure 2:**
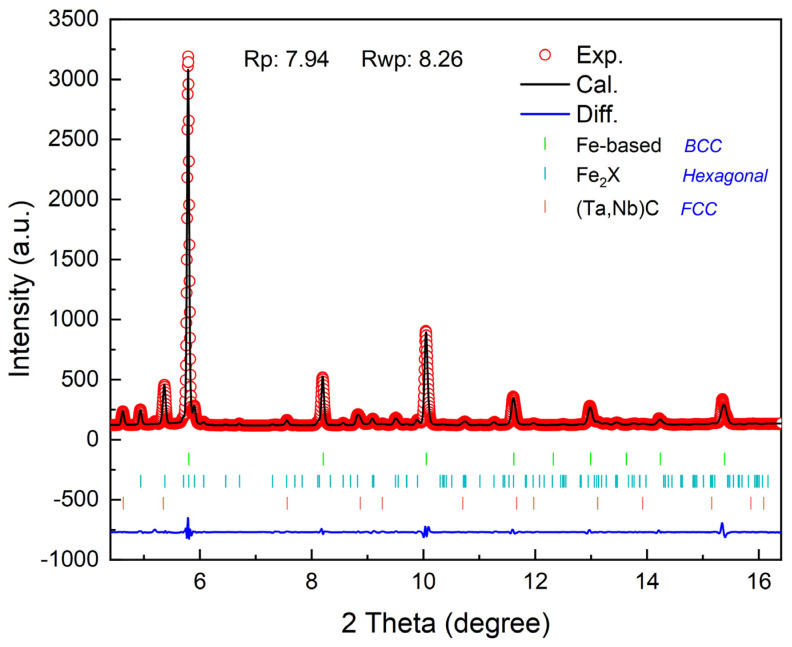
High–energy synchrotron X-ray diffraction pattern and refinement result of laser–cladding WMoTaNb/316L coating.

The morphology and X-ray diffraction pattern of the laser-cladding powders are shown in Figure 1. The 316L stainless steel and WMoTaNb powders exhibit single-phase FCC and BCC structures, respectively, with good sphericity, which is conducive to improving the flowability of the powders. Figure 2 shows the S-XRD patterns and refinement results of the laser-cladding WMoTaNb/316L composite coating, which consist of an Fe-based solid solution with a BCC structure, intermetallic compounds (IMCs) with hexagonal structure, and carbides with an FCC structure. Moreover, the experimental data indicated by the red circle, while the refinement data is represented by the black line. These IMCs with a hexagonal structure are well matched with diffraction peaks of the Fe_2_W-type Laves phase (PDF# 97-063-4064) [38]. However, these IMCs are enriched with refractory elements such as W, Mo, Ta, and Nb in addition to Fe in this work. This may be attributed to the fact that these elements exhibit the same crystal structure and small differences in atomic radii, which leads to the formation of the Laves phase containing multiple elements [39]. Therefore, the IMCs were identified as the Fe_2_X (X = W, Mo, Ta, Nb) Laves phase. In addition, a (Ta, Nb)C FCC phase is also present in the coatings (PDF# 51-1421), and the corresponding angle of the (111) crystal plane of the (Ta, Nb)C phase is 4.63°. According to the Bragg equation, its interplanar spacing is calculated to be 2.550 Å. After refining the experimental data, the phase fractions of the three phases in the composite coating are 72.7 wt.% (Fe-based solid solution), 23.4 wt.% (Fe_2_X (X = W, Mo, Ta, Nb)), and 3.9 wt.% ((Ta, Nb)C) each.

### 3.2. Microstructures and Evolutions

**Figure 3:**
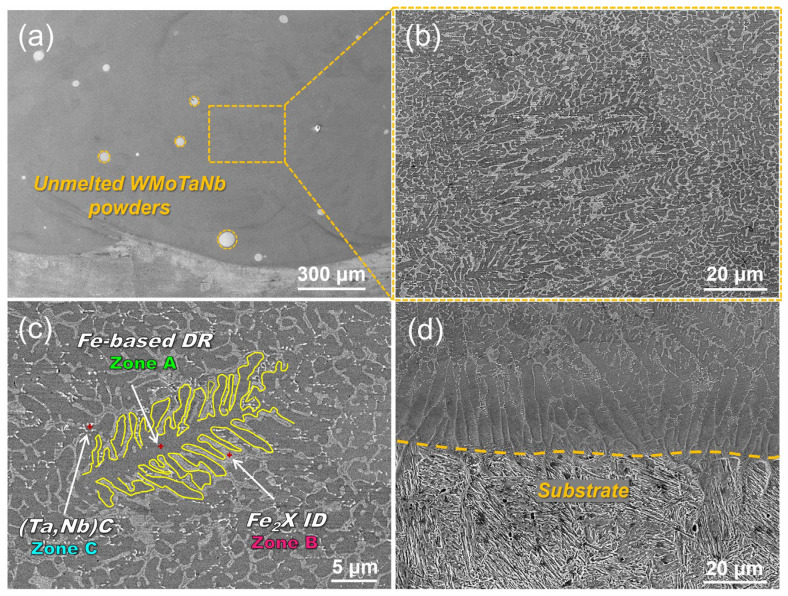
SEM images of cross-sectional laser-cladding WMoTaNb/316L coating: (**a**) macroscopic morphology, (**b**) microscopic morphology of the coating in the region marked in (**a**,**c**) microscopic morphology at a larger magnification, and (**d**) microscopic morphology of the bond interface between the cladding coating and the substrate.

Figure 3a shows the SEM image of the overall structure of the crosssection of the laser-cladding WMoTaNb/316L composite coating. An excellent metallurgical bonding without cracks or porosity between the cladding coating and the substrate has been achieved. However, due to the high melting point of WMoTaNb RHEA, few incompletely melted WMoTaNb powders still exist in the coatings. Figure 3b shows the microstructure evolution morphology of the coating in the region marked in Figure 3a, and the morphology at a higher magnification is illustrated in Figure 3c. It can be observed that the whole coating consists of a gray network inter-dendritic (ID) phase (zone A), dark gray dendritic (DR) or columnar dendrites (zone B), and a fine diffuse white granular phase (zone C). According to the EDS results (shown in Table 2) and the above analysis, it is known that the gray network phase (point A) is the Fe_2_X (Wo, Mo, Ta, Nb) hexagonal phase and the dark gray dendritic or columnar crystals (point B) is the Fe-based BCC phase. The white granular phase (point C) is the FCC (Ta, Nb) C phase. However, the resolution of EDS for ultralight elements (O, N, C, etc.) is insufficient, making it challenging to perform a quantitative analysis on the C element. Therefore, only qualitative illustration is possible, which is explained in more detail later in this article.

The microstructures of the interface between the coatings and substrate, as depicted in Figure 3d, exhibit highly oriented planar crystals growing perpendicular to the substrate direction due to obligatory growth resulting from different temperature gradients and solidification rates within the molten pool. According to the theory of solidification supercooling, crystal growth direction and morphology during rapid solidification of laser-cladding coatings are influenced by temperature gradient (G), solidification rate (R), and their ratio G/R [40]. The substrate and powders are rapidly melted by the laser, forming a molten pool that quickly solidifies upon the movement of the laser. The maximum growth rate occurs at the bottom of the melt pool, forming planar crystals and epitaxial growth [41]. As depicted in Figure 3d, the solidification rate increases while the temperature gradient decreases during the advancement of the solid-liquid interface during solidification, leading to a transition in grain morphology from planar crystals to columnar dendrites. The primary factor contributing to the growth of columnar dendrites in alloys under positive temperature gradients is compositional subcooling occurring along the solid–liquid interface front during this process. The laser-cladding process involves rapid heating and cooling, which is a typical non-equilibrium solidification process. During this process, the concentration distribution of solutes in the liquid phase inevitably changes, resulting in compositional subcooling. The region of compositional subcooling increases as the G/R ratio decreases. According to the solidification theory, compositional subcooling disrupts the planar growth state of the solid–liquid interface, inducing the protrusion of bumps into the subcooled liquid phase. This phenomenon accelerates the growth rate of the interface and facilitates its further growth into the liquid. Finally, columnar dendrites are formed during rapid cooling [42]. As depicted in Figure 3b, the temperature gradient (G) continues to decrease and the solidification rate (R) continues to increase in the middle region of the coating, resulting in a further decrease in G/R. This transformation leads to fine dendritic structures from the coating’s dark gray coarse columnar dendrites. The microstructure of the coating mainly comprises thicker columnar dendrites, finer dendrites, and inter-dendritic reticulation.

**Figure 4:**
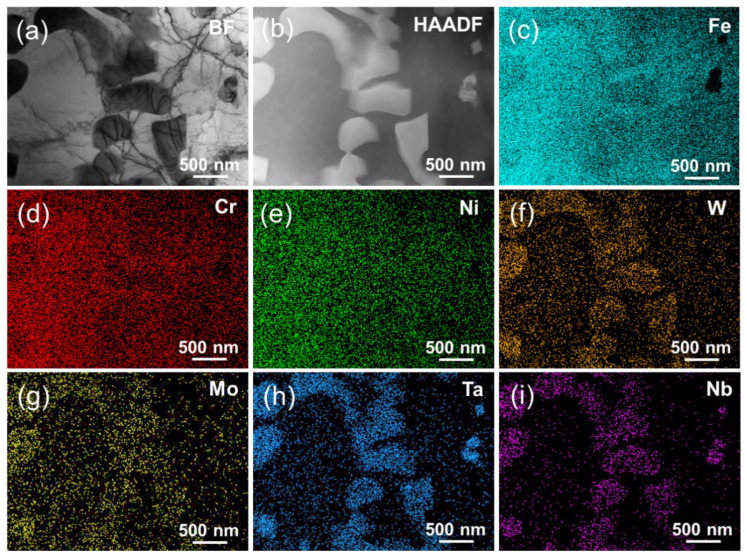
TEM and STEM–HAADF images of laser-cladding WMoTaNb/316L composite coating surface. (**a**) TEM–BF image; (**b**) STEM–HAADF image; and elemental distribution of (**c**) Fe, (**d**) Cr, (**e**) Ni, (**f**) W, (**g**) Mo, (**h**) Ta, (**i**) Nb.

**Figure 5:**
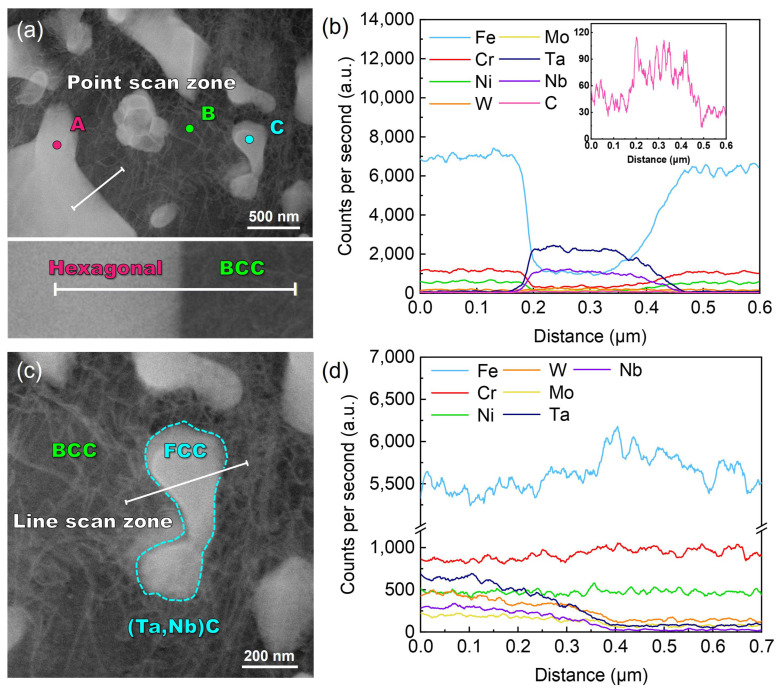
STEM–HAADF images and EDS-line scan of laser-cladding WMoTaNb/316L composite coating surface: (**a**,**b**) line scan zone between hexagonal and BCC phases and compositional variations, (**c**,**d**) line scan zone between FCC and BCC phases and compositional variations.

Figure 4 shows the bright field transmission electron microscopy (BF TEM), high angle annular dark field-scanning transmission electron microscopy (HAADF-STEM), and elemental distribution of the laser-cladding WMoTaNb/316L composite coating. The combination of XRD, SEM, and EDS results indicates that the white area observed in the BF image corresponds to the Fe-based solid solution phase present in the dendrites. The scanning area is covered mainly by the three elements Fe, Cr, and Ni, with the Fe element being relatively more enriched in the dendrites (Figure 4c). This suggests that Fe elements in 316L stainless steel and the substrate are involved in the formation of the entire coating during the high power laser-cladding process. As a result, a large amount of Fe-based BCC solid solution was formed in the coating under this high-energy laser-cladding. The elemental mapping distribution in Figure 4 and the elemental line scan in Figure 5a,b reveal distinct segregation of refractory elements, namely W, Mo, Ta, and Nb, among the inter-dendritic structures. Additionally, an increase in Fe content is observed, while a decrease in refractory element content is observed.

Furthermore, both Cr and Ni from 316L exhibit equal distribution between the two phases. The elements W, Mo, Ta, Nb, and Fe form a hexagonal Laves phase structure, visually representing a black inter-dendritic phase in the TEM-BF image. In addition, the comparison of the elemental mapping distribution in Figure 4c–i reveals distinct regions with low Fe content and high Ta and Nb concentrations. The granular phase observed is believed to be the in situ formation of the (Ta, Nb)C FCC phase between Ta, Nb, and C during cladding. As evident from the elemental line scans in Figure 5c,d, there is a significant increase in Ta and Nb elemental signal intensity when passing through the FCC granular phase. Although the C element exhibits limited production of characteristic X-rays due to its small number of orbital electrons, resulting in a low acquired signal intensity [43], the distribution of C in this region can be observed by analyzing the trend of signal intensity change illustrated in Figure 5d. The area marked by orange dots in Figure 5a was subjected to an EDS-point scan, and a quantitative analysis of the element content of the three phases was conducted. The corresponding results are presented in Table 3. Next, the phase structure of the laser-cladding coatings was further characterized using selected electron diffraction mode (SEAD).

**Figure 6:**
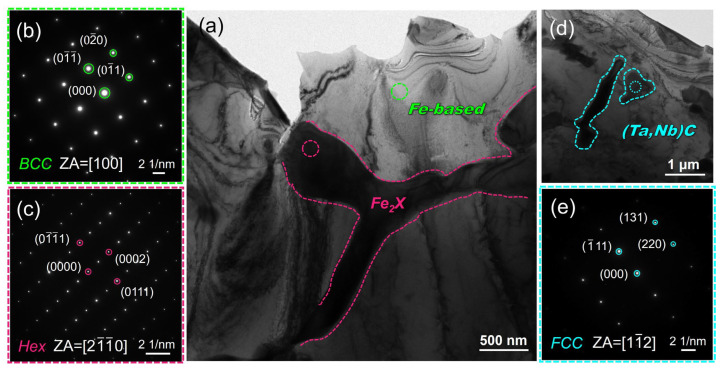
TEM bright–field micrographs and SAED of Fe–based solid solution and Fe_2_X (W, Mo, Ta, Nb) Laves phase. (**a**) BF image, (**b**) and (**c**) SAED patterns of Fe–based solid solution and Fe_2_X phases shown in (**a**), and (**d**) and (**e**) BF image and SAED patterns of (Ta, Nb)C phase.

The coatings were characterized using TEM-SAED to analyze the phase structure and orientation relationship further. Figure 6a clearly shows distinct white zones and black structures, corresponding, respectively, to the dendritic and inter-dendritic network phases observed in Figure 3c. The SAED analysis results of the red-circled zone in Figure 6a depicted in Figure 6b confirm that the crystal structure of the predominant Fe-based dendritic phase in the coating is BCC, exhibiting a (110) crystal face spacing of approximately 2.139 Å, which aligns well with the XRD findings. The results indicate that the crystal face spacing of Fe-based dendritic phases is slightly larger than that of the α-Fe (110) crystal face [44]. This can be primarily attributed to the partial dissolution of refractory elements such as W, Mo, Ta, and Nb with larger atomic radii compared to Fe. As a result, Fe-based dendritic phases exhibit increased crystal face spacing and cell constants [45]. Moreover, Figure 6d illustrates the morphology of the (Ta, Nb)C phase in a bright field image, exhibiting a rod-like or granular shape, which can be easily distinguished from the Fe_2_X Laves phase through STEM-HADDF EDS mapping. The SAED pattern of the site indicated by the circle in Figure 6d is presented in Figure 6e, confirming the identification of the phase as the (Ta, Nb)C phase with an FCC structure. The crystal plane spacing of (111) is determined to be 2.611 Å, which also agrees well with the XRD results. In conclusion, the combined XRD, EDS, and TEM-SAED results unequivocally ascertain the presence of an Fe-based solid solution, Fe_2_X(W, Mo, Ta, Nb), as well as (Ta, Nb)C in the coatings.

**Figure 7:**
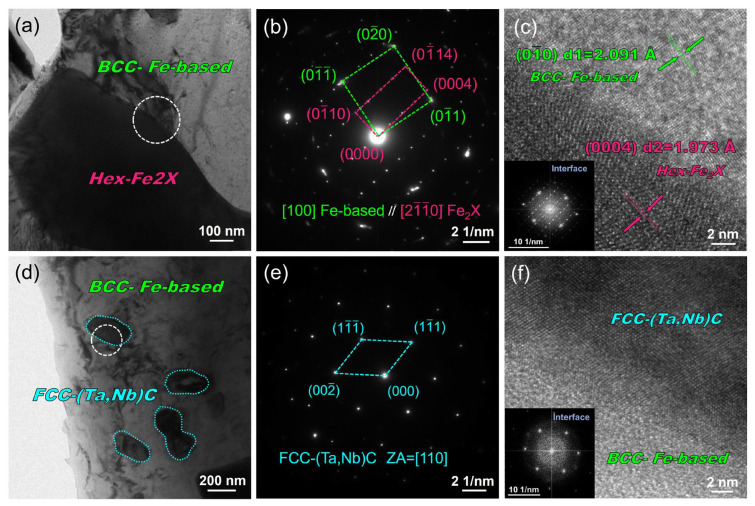
TEM–BF micrograph, SAED pattern, and HRTEM image at the interface. (**a**–**c**) Between Fe-based BCC solid solution and Fe_2_X (W, Mo, Ta, Nb) Laves phase, and (**d**–**f**) between Fe-based BCC solid solution and (Ta, Nb)C phase.

The SAED pattern in Figure 7b illustrates the interface between the Fe_2_X Laves phase and the Fe-based solid solution, which was obtained from the region marked by the white dashed circle in the TEM-BF image shown in Figure 7a. The SAED pattern reveals the coexistence of hexagonal and BCC structures in the selected region, corresponding to the band axes of [2$\overset{-}{1}\overset{-}{1}$0] and [100], respectively. The approximate Fe_2_X phase and Fe-based solid solution orientations are <2$\overset{-}{1}\overset{-}{1}$0>*_Fe__2X_*|| <110>*_Fe-based_* and {0004}*_Fe__2X_*||{0$\overset{-}{1}$0}*_Fe-based_*. The degree of co-gridding at the two-phase interface is related to the degree of mismatch [46]. δ is the relative difference in atomic spacing between two neighboring phases at the interface [47]:

(2) $$\delta =\frac{2\left(d_{1}-d_{2}\right)}{d_{1}+d_{2}}$$

where *d*_1_ and *d*_2_ are the plane spacings of the Fe-based solid solution/Fe_2_X Laves phase, respectively. Measurements according to Figure 7b yielded the following data: 2.091 Å for d(0$\overset{-}{1}$0)*_Fe-based_* and 1.973 Å for d(0004)*_Fe__2X_*. From Equation (1), the |*δ*| value is 5.81%. This can be regarded as a semi-coherent interface between the two phases, exhibiting significantly lower chemical energy than the incoherent interface due to partial atomic matching. In addition, the (Ta, Nb)C phase and the Fe-based solid solution do not exhibit a clearly defined orientation relationship. The SAED pattern and high-resolution transmission electron microscope (HRTEM) image in Figure 7e,f depict the interface between the Fe-based solid solution and the (Ta, Nb)C phase (highlighted by the white circle in Figure 7d). Specifically, only the diffraction pattern of the (Ta, Nb)C phase is observable along the [110] crystallographic band axis. In contrast, the HRTEM image reveals poor resolution for the BCC Fe-based solid solution region (as shown in Figure 7f).

**Figure 8:**
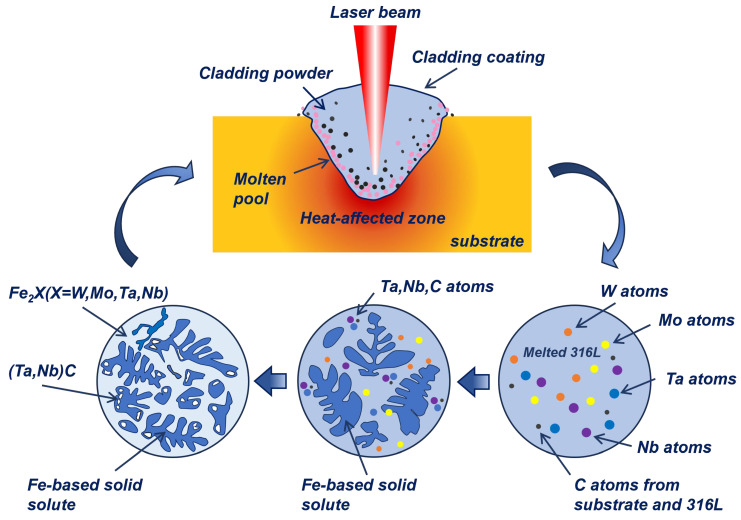
Schematic of the melting–solidification process during laser-cladding.

A schematic of the melting-solidification process during laser-cladding is illustrated in Figure 8 to understand the solidification process better. During the cladding process, the 316L feedstock and substrate are first melted under a laser beam due to the high temperature, and a melt pool is formed. The molten pool solidified as soon as the laser beam left the molten pool. Based on the aforementioned SEM results, the dendritic growth of the Fe-based solid solution with a BCC structure is evident in Figure 3c. At the same time, the Laves phases are dispersed among the inter-dendrites, primarily due to element segregation during the solidification process (as depicted in Figure 5b). The refractory elements W, Mo, Ta, and Nb have significantly larger atomic radii compared to other elements, resulting in poor mutual solubility with Fe, Cr, and Ni during solidification. The refractory elements are thus readily excluded from the forefront of the solid–liquid interface, resulting in dendritic segregation. Furthermore, due to their negative enthalpies of mixing with other elements (as shown in Table 4), these refractory elements exhibit limited solubility within the Fe-based solid solution lattice and, therefore, tend to form intermetallic compounds [48]. The crystal structures of Fe_2_W, Fe_2_Mo, Fe_2_Ta, and Fe_2_Nb exhibit an identical hexagonal structure with the same space group P63/MMC, and the differences in lattice constants are negligible. The formation of an Fe_2_X (W, Mo, Ta, Nb) Laves phase through a mutual solid solution between W, Mo, Ta, and Nb is therefore readily achievable. Additionally, it is evident from Figure 3c that the precipitation of (Ta, Nb)C particles primarily occurs along the inter-dendritic edges of the Fe-based solid solution, indicating that NbC forms during the final stage of solidification [49]. The formation of (Ta, Nb)C is mainly attributed to the strong affinity of Ta and Nb for C, and the similarity of atomic radii and chemical properties of Ta and Nb. In addition, the cladding of subsequent layers during the multi-pass laser-cladding process can induce thermal effects on the solidified regions. The aging treatment involving multiple thermal cycles induces the outward diffusion of solute atoms in the supersaturated Fe-based solid solution forming from rapid cooling [38], thereby further facilitating the formation of an Fe_2_X Laves phase and (Ta, Nb)C carbide.

### 3.3. Microhardness

**Figure 9:**
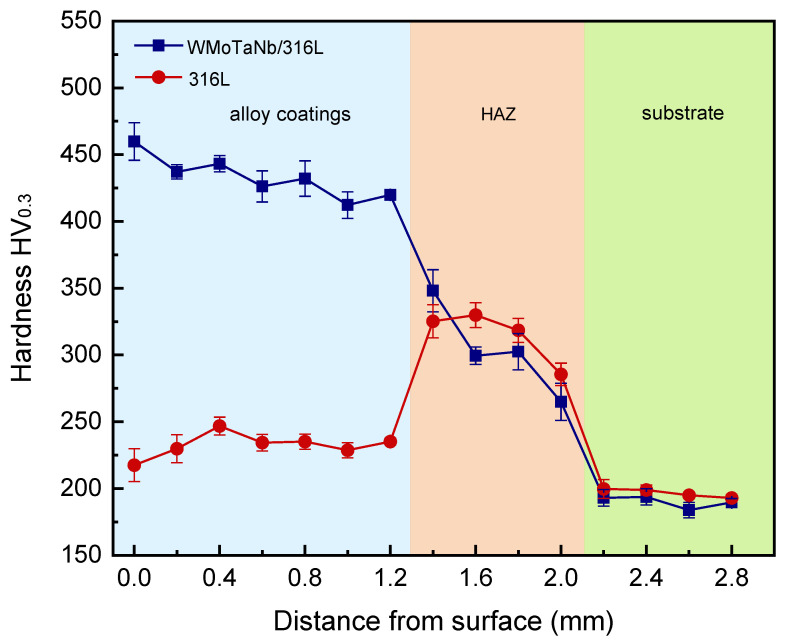
Microhardness of the laser-cladding WMoTaNb/316L coating and laser-cladding 316L coating.

Figure 9 shows the microhardness distribution of the WMoTaNb/316L stainless steel composite coating from the surface to the substrate. The 316L stainless steel coating is also included for benchmarking. Based on the cross-sectional morphology, the hardness can be categorized into three zones—the WMoTaNb/316L composite coating zone, the heat-affected zone (HAZ), and the 45 steel substrate zone—each exhibiting distinct hardness levels. The surface hardness of ~460 HV_0.3_ is significantly higher than that of the substrate. Furthermore, it exhibits a 2.1-fold increase compared to the surface hardness of the laser-cladding 316L coating. It can also be seen that the hardness of the composite coating tends to decrease from the surface to the substrate. The possible cause for this phenomenon could be attributed to the alteration in the microstructure of the coating. The dendrites near the surface exhibit a finer morphology compared to the coarser dendrites observed at the bottom of the coating. Finally, during the laser-cladding process, the substrate inevitably dilutes the coating, resulting in higher dilution and lower hardness in regions closer to the substrate. These two mechanisms contribute to the observed trend of variation in coating cross-sectional hardness.

**Figure 10:**
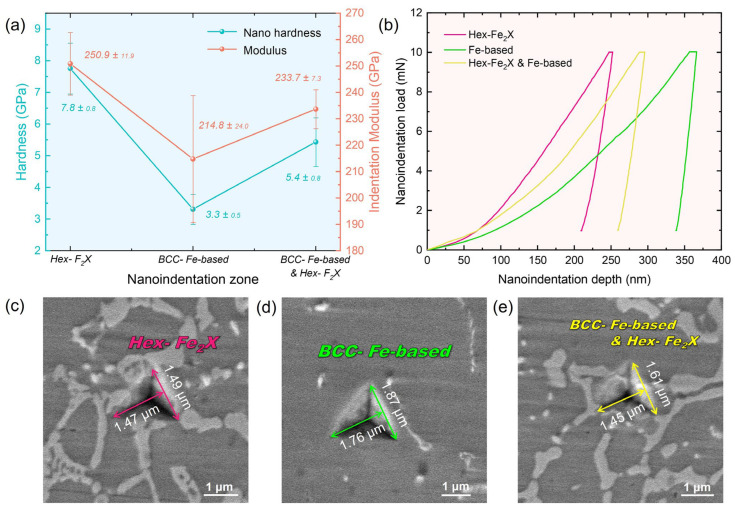
The nanoindentation behavior of the Fe_2_X (W, Mo, Ta, Nb) Laves phase, Fe-based solid solution, and the common zone between them in the WMoTaNb/316L composite coating. (**a**) Hardness and elastic modulus. (**b**) Representative load–displacement curves. Representative nanoindentation zone SEM of (**c**) Fe_2_X (W, Mo, Ta, Nb) Laves phase, (**d**) Fe-based solid solution, and (**e**) the common zone of Fe_2_X (W, Mo, Ta, Nb) Laves phase and Fe-based solid solution.

The strengthening mechanisms of metal materials primarily include dislocation strengthening, solid solution strengthening, second phase strengthening, and fine crystal strengthening. For instance, in accordance with the Hall–Petch relationship [52], a decrease in average grain diameter leads to an increase in material yield strength. The hardness enhancement of a WMoTaNb/316L coating is mainly attributed to the distribution of Fe_2_X (W, Mo, Ta, and Nb) Laves phases among the dendrites. These Laves phases have a topologically closed phase (TCP) structure with high coordination number, space-filling degree, large unit cell, and dislocation vectors, which inhibit the dislocation nucleation and slip [53]. In addition, the Laves phase also has unique advantages in the field of energy materials. For example, the Laves-phase HEAs are usually used for hydrogen storage or Ni-MH battery applications [54,55,56,57,58]. Figure 10 illustrates the results of nanoindentation experiments conducted across distinct zones of the WMoTaNb/316L composite coating. Specifically, Figure 10a shows the respective hardness and modulus values of the Fe_2_X (W, Mo, Ta, Nb) Laves phase, Fe-based solid solution, and the common zone between them. The representative test zone corresponding to the results depicted in Figure 10c–e. The experiment results reveal that the Fe_2_X Laves phase exhibits significantly higher values for hardness and elastic modulus, measuring 7.8 ± 0.8 GPa and 250.9 ± 11.9 GPa, respectively, compared to those of the Fe-based solid solution (hardness: 3.3 ± 0.5 GPa; elastic modulus: 214.75 ± 24.0 GPa). The experimental results of the common zone of Fe_2_X Laves phase and Fe-based solid solution (hardness: 5.4 ± 0.8 GPa, elastic modulus: 233.7 ± 7.3 GPa) are also higher than the Fe-based solid solution. The presence of the Fe_2_X Laves phase significantly enhances the deformation resistance of the composite coating, as clearly demonstrated by its combination with the representative load–displacement curves depicted in Figure 10b. The indentation depth of the common zone in the Fe_2_X Laves phase and Fe-based solid solution is smaller than that in Fe-based solid solution under identical loading conditions. Therefore, the densely distributed Laves inter-dendritic phase is anticipated to play a crucial role in enhancing the coating hardness.

**Figure 11:**
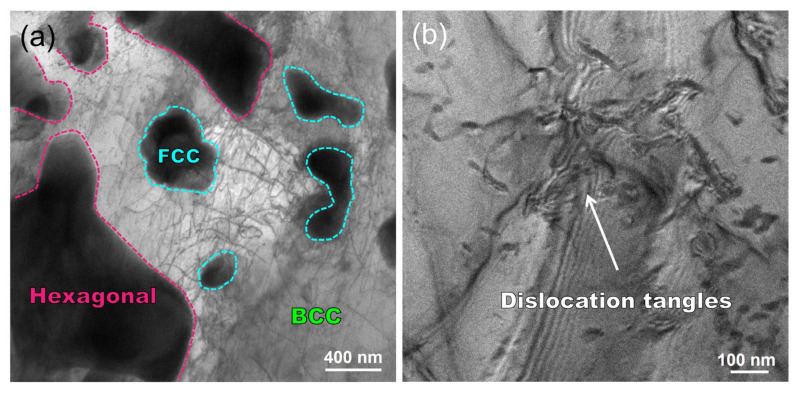
TEM bright–field micrographs of typical microstructures of WMoTaNb/316L laser-cladding composite coating: (**a**) dislocation distribution; (**b**) evidence of dislocation entanglement.

The second point is that Figure 11a clearly illustrates a significant presence of dislocations within the BCC Fe-based solid solution phase. As shown in Figure 11b, the close examination of the Fe-based solid solution phase reveals the presence of dislocation entanglements. The additive manufacturing process is believed to induce substantial plastic strain due to post-solidification thermal expansion and contraction, as well as rapid localized heating and cooling cycles, resulting in a high dislocation density [59,60]. The interaction among a significant number of dislocations impedes the dislocation motion, thereby promoting the hardness of the coating. Moreover, the presence of trace elements such as tungsten and molybdenum dissolved in the BCC Fe-based solid solution contributes to the strengthening of the coating through solid solution strengthening mechanisms. Consequently, this also enhances the hardness properties of the coating.

### 3.4. Wear Resistance

**Figure 12:**
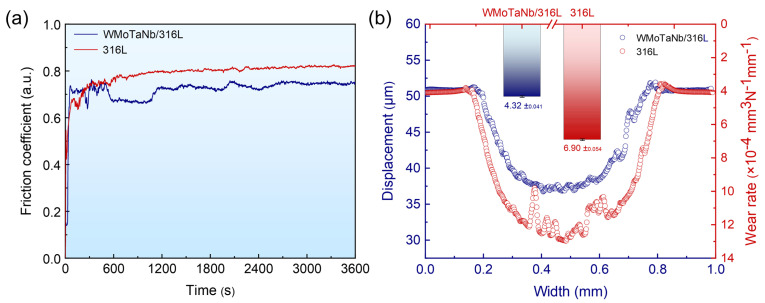
The wear resistance comparison between laser-cladding WMoTaNb/316L coating and 316L coating. (**a**) Friction coefficients. (**b**) Profiles of wear grooves and wear rates.

Figure 12a shows the friction coefficient curves of laser-cladding WMoTaNb/316L composite coating and 316L coating. The friction coefficient curve of the composite coating exhibits significant fluctuations following the occurrence of friction. At the early stage of wear, the Al_2_O_3_ ceramic ball rubs the surface of the coating and produces point contact friction. As the contact area between the friction partner and the coating surface gradually increases, it enters the intense wear stage. The friction coefficient tends to stabilize and enter the stable wear stage as the friction process progresses [61]. The friction coefficient of the composite coating ranges from 0.65 to 0.75. In contrast, the friction coefficient of the 316L coating exhibits a relatively gradual change and stabilizes at approximately 0.82 upon entering the stage of stable wear.

**Figure 13:**
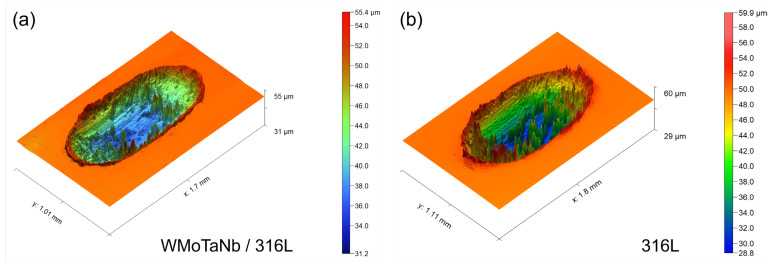
Three-dimensional morphologies of the worn surface track in (**a**) WMoTaNb/316L composite coating and (**b**) 316L coating.

The cross-sectional profile of the wear grooves in Figure 12b reveals that the WMoTaNb/316L composite coating exhibits a shallower grooves depth, narrower width, and smoother profile line. The three-dimensional (3D) morphologies of the wear surface track in Figure 13 clearly shows that the 316L coating has a rougher wear surface, indicating more generation of wear chips during friction and intensifying the wear behavior. Moreover, the wear rate of the coatings is illustrated in Figure 12b, wherein the wear rate of WMoTaNb/316L composite coating (4.32 × 10^−4^ mm^3^ N^−1^ mm^−1^) exhibits a significantly lower value compared to that of the 316L coating (6.90 × 10^−4^ mm^3^ N^−1^ mm^−1^). According to Archard’s tribological theory [62], there is a proportional relationship between the hardness of a coating and its wear resistance. The reduction in the coefficient of friction of the coating is mainly due to the improvement in hardness.

**Figure 14:**
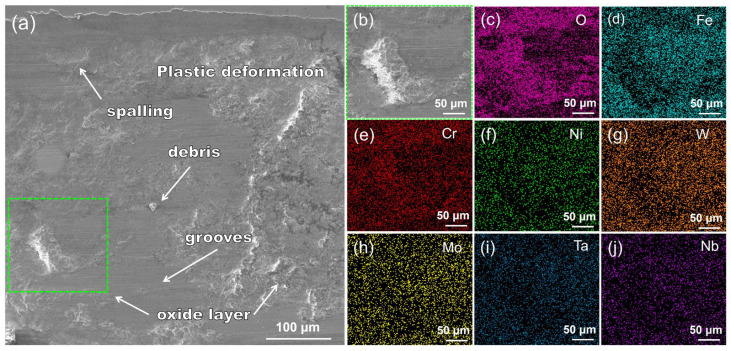
SEM images and EDS elemental distribution of worn morphology of (**a**) WMoTaNb/316L composite coating surface; (**b**) enlarged view of the green box in (**a**) and elemental distribution of (**c**) O, (**d**) Fe, (**e**) Cr, (**f**) Ni, (**g**) W, (**h**) Mo, (**i**) Ta, and (**j**) Nb.

Figure 14a shows the worn morphology of the WMoTaNb/316L composite coating. The surface of the coating exhibits a relatively rough texture, characterized by plastic deformation, flaking pits, wear debris, and grooves. This is because a large number of micro-convexities still exist on the microscopic surface after polishing the coating surface. When the friction partner comes into contact with the micro-convexities of the coating surface and applies pressure, plastic deformation and adhesion occur [63]. In the process of sliding, the adhered material is sheared and damaged, shedding to form abrasive particles, causing abrasive wear and creating grooves on the coating surface [64]. The green boxed area in Figure 14a was subjected to EDS elemental distribution analysis, revealing localized bias towards O and Cr elements. This may be attributed to the frictional heat generated during the sliding process, leading to the oxidation of the adhesive material. However, it can be observed that the oxide layer lacks continuity and tends to be dislodged during repeated friction, which in turn forms oxidized debris and aggravates abrasive wear. In summary, the main wear mechanisms observed in this study were adhesive wear, oxidative wear, and abrasive wear.

## 4. Conclusions

The present work successfully prepared the WMoTaNb/316L composite coatings with no obvious defects and good metallurgical bonding with the substrate. The microstructures of the composite coatings were systematically characterized. The microhardness and wear resistance were analyzed in comparison with those of the laser-cladding 316L coatings. The main conclusions are as follows:

1. The microstructure of the coating consists of a BCC Fe-based dendritic solid solution, a hexagonal Fe_2_X (W, Mo, Ta, and Nb) Laves interdendritic phase, and an FCC (Ta, Nb)C interdendritic granular phase.
2. The Fe-based solid solution and the Fe_2_X Laves phase show a semi-coherent interface with a lattice mismatch of 5.81%. There is no orientation relationship between the Fe-based solid solution and the (Ta, Nb)C phase, which is a non-coherent interface.
3. The microhardness of the laser-cladding WMoTaNb/316L composite coating surface was about 460 HV_0.3_, which was 2.1 times higher than that of the 316L coating. The primary strengthening mechanism involves the incorporation of a WMoTaNb refractory high entropy alloy, which facilitates the formation of a robust network Fe_2_X Laves phase with enhanced strength, as well as solid solution strengthening within the iron-based solid solution.
4. The wear resistance of laser-cladding WMoTaNb/316L composite coatings is superior to that of 316L coatings. The main wear mechanisms at room temperature were adhesive, oxidative, and abrasive wear.

## Figures and Tables

**Table 1:** Chemical composition of laser-cladding powders and 45 steel substrate (wt.%).

Elements	W	Mo	Ta	Nb	Fe	Cr	Ni	Si	Mn	C	S	P	O
WMoTaNb	21.00	26.00	24.00	29.00	-	-	-	-	-	-	-	-	-
316L	-	2.59	-	-	Bal.	16.56	10.75	0.31	0.68	0.015	0.005	0.012	0.07
Substrate	-	-	-	-	Bal.	0.02	0.01	0.20	0.53	0.45	0.009	0.021	-

**Table 2:** EDS results of each zone shown in Figure 3 (at. %).

Elements	W	Mo	Ta	Nb	Fe	Cr	Ni
Zone A	1.7	2.3	0.4	0.2	76.0	11.8	7.6
Zone B	5.3	5.6	7.2	6.4	60.6	9.9	5.0
Zone C	4.9	5.2	22.0	24.5	31.8	7.7	3.8

**Table 3:** EDS results of each zone shown in Figure 5 (at.%).

Elements	W	Mo	Ta	Nb	Fe	Cr	Ni	C
Point A	4.4	5.3	6.2	6.2	63.1	10.0	4.8	-
Point B	1.5	2.3	0.3	0.4	76.4	13.2	5.9	-
Point C	1.5	2.7	32.1	33.2	13.3	3.6	0.8	12.8

**Table 4:** The values of mixing enthalpy of element pairs and atomic radius [50,51].

Element	Atomic Radius (Å)	∆Hmix (kJ∙mol^−1^)						
		Fe	Cr	Ni	W	Mo	Ta	Nb
Fe	1.27	-	−1	−2	0	−2	−15	−16
Cr	1.28		-	−7	1	0	−7	−7
Ni	1.24			-	−3	−7	−29	−30
W	1.41				-	0	−7	−8
Mo	1.40					-	−5	−6
Ta	1.47						-	0
Nb	1.48							-

## Data Availability

The original contributions presented in the study are included in the article, further inquiries can be directed to the corresponding authors.
